# Nicotine Withdrawal Drives Aversive Behaviors by Recruiting Inhibitory Interpeduncular Nucleus Inputs to the Laterodorsal Tegmentum in Mice

**DOI:** 10.1523/JNEUROSCI.2405-24.2025

**Published:** 2025-07-14

**Authors:** Alexis Monical, Daniel S. McGehee

**Affiliations:** ^1^Medical Scientist Training Program, University of Chicago, Chicago, Illinois 60637; ^2^Department of Anesthesia and Critical Care, University of Chicago, Chicago, Illinois 60637

**Keywords:** acetylcholine, addiction, dopamine, fiber photometry, GABA, optogenetics

## Abstract

Nicotine addiction remains a major cause of disease and premature death worldwide. Nicotine modulates neural pathways that underlie both rewarding and aversive behavioral effects, but persistent activation of brain reward circuitry drives nicotine consumption despite the negative consequences. When nicotine users attempt to quit, additional neural mechanisms are recruited to generate an aversive withdrawal state, which contributes to the remarkably high relapse rate among nicotine users. The interpeduncular nucleus (IPN) and its presynaptic inputs from the medial habenula are key mediators of aversive nicotine withdrawal symptoms, but the downstream neural targets mediating these effects are unknown. The aversive effects of acute exposure to high doses of nicotine require inhibitory IPN GABAergic projections to the laterodorsal tegmentum (LDTg), a key driver of reward-related dopamine signaling. Here we show that optogenetic inhibition of these IPN→LDTg projections reduces behavioral and physiological effects of nicotine withdrawal in male and female mice. Using fiber photometry, we found that nicotine withdrawal reduced reward-related signaling with decreases in both LDTg neuronal activity and nucleus accumbens dopamine release. These studies demonstrate a direct link between aversive and appetitive neural pathways that is active during nicotine withdrawal, providing novel targets for treating nicotine addiction.

## Significance Statement

The likelihood of relapse to drug taking is higher for nicotine than all other addictive drugs. While the rewarding effects of the drug and strong cue associations are known to contribute to relapse, avoidance or reduction of withdrawal symptoms is a major factor driving individuals back to nicotine consumption. Indeed, one of the key features of available treatments for nicotine abuse is amelioration of withdrawal signs. Therefore, understanding the neural mechanisms underlying the symptoms of nicotine withdrawal is critical for identifying new treatment strategies. Here we provide compelling evidence that inhibitory projections from the interpeduncular nucleus to the laterodorsal tegmental nucleus are an essential mediator of the symptoms of nicotine withdrawal.

## Introduction

Nicotine addiction affects over 1 billion people worldwide and the associated adverse health consequences create a severe threat to public health (Samet, 2013). A key element in the rewarding effects of nicotine is the excitation of ventral tegmental area (VTA) dopaminergic projections to the nucleus accumbens (NAc; Di Chiara and Imperato, 1988; Corrigall et al., 1992; Fu et al., 2000; Suto et al., 2001; Mansvelder et al., 2002; Mao et al., 2011). A major challenge in overcoming nicotine addiction is the remarkably high relapse rate for individuals who attempt to quit (Chaiton et al., 2016). An important driver of relapse is avoidance of the aversive effects associated with nicotine withdrawal, which are characterized by physical and psychological symptoms including anxiety, depression, irritability, and restlessness (Jackson et al., 2015; McLaughlin et al., 2015). During withdrawal from chronic nicotine, tonic dopamine (DA) levels within the NAc are reduced, which raises reward thresholds contributing to depression-like symptoms (Epping-Jordan et al., 1998; Grieder et al., 2012; Zhang et al., 2012; Baiamonte et al., 2014). Understanding the neural mechanisms mediating nicotine withdrawal is essential for developing improved therapeutics.

A primary mechanism underlying nicotine withdrawal is activation of the interpeduncular nucleus (IPN), a midbrain GABAergic center, that mediates the associated cognitive and behavioral effects (Antolin-Fontes et al., 2015; Molas et al., 2017a). Withdrawal from chronic nicotine activates IPN GABAergic neurons through recruitment of excitatory inputs from the medial habenula (MHb), and this activation is associated with both somatic and affective withdrawal symptoms (Salas et al., 2009; Zhao-Shea et al., 2013, 2015). Despite the evidence implicating the IPN in nicotine withdrawal, the downstream projections from IPN that mediate nicotine withdrawal symptoms are currently unknown.

Neuroanatomical tracing studies reveal synaptic connections between the IPN and the laterodorsal tegmental nucleus (LDTg; Quina et al., 2017; Bueno et al., 2019), a brainstem structure that modulates reward processing and motivation via acetylcholine, GABA, and glutamate projections to the mesolimbic DA system (Wang and Morales, 2009; Steidl and Veverka, 2015; Dautan et al., 2016; Coimbra et al., 2019; Liu et al., 2022). Recent studies reported that the LDTg is activated by rewarding concentrations of nicotine in ex vivo brain slice recording and that lesions of the LDTg in vivo reduce the locomotor effects of nicotine (Alderson et al., 2005; Ishibashi et al., 2009). We previously reported IPN GABAergic projections to the LDTg contributed to an aversive state following high-dose nicotine administration and that optogenetic inhibition of these projections blocks conditioned place aversion to high-dose nicotine (Wolfman et al., 2018). Inhibiting the IPN projections to the LDTg also reduced anxiety-associated behavior (Wolfman et al., 2018). Interestingly, both acute high-dose nicotine and withdrawal from chronic nicotine recruit the MHb→IPN pathway to produce an aversive behavioral state (Fowler et al., 2011; Zhao-Shea et al., 2013; Antolin-Fontes et al., 2015). These results suggest that IPN inhibition of LDTg neurons suppresses mesoaccumbens DA levels during nicotine withdrawal to mediate the associated aversive effects.

LDTg neurons synapse onto VTA DA neurons that project to the NAc (Oakman et al., 1995; Omelchenko and Sesack, 2005; Coimbra et al., 2017). Excitatory cholinergic and glutamatergic drive from the LDTg to the VTA is a critical regulator of DA neuron activity, which gates the transition of DA neurons to burst activity, to mediate conditioned place preference and reinforcement of operant behaviors (Blaha et al., 1996; Forster and Blaha, 2000; Lodge and Grace, 2006; Lammel et al., 2012; Chen and Lodge, 2013; Xiao et al., 2016; Coimbra et al., 2017; Steidl et al., 2017). Additionally, inhibitory LDTg GABAergic neurons project to the VTA (Broussot et al., 2022; Liu et al., 2022) and are activated along with glutamatergic neurons in response to nicotine (Ishibashi et al., 2009). Here we have investigated the role of the IPN→LDTg connections in nicotine withdrawal, using a combination of behavior, fiber photometry, and optogenetic approaches to monitor and manipulate this circuitry.

## Materials and Methods

### Animals

All experiments were done in compliance with the animal care guidelines set forth by the National Institutes of Health and were approved by the University of Chicago's Institutional Animal Care and Use Committee. Mice were group housed (2–5 mice/cage) on a standard light/dark cycle (6 A.M.–6 P.M.) with experimentation during the light cycle. The following mouse strains (C57BL/6J background, backcrossed for at least 10 generations) were used: C57BL/6J (JAX stock number: 000664), Gad2-IRES-Cre (JAX stock number: 028867), Vglut2-IRES-Cre (JAX stock number: 028863), and ChAT-IRES-Cre (JAX stock number: 006410). Water and standard chow were available *ad libitum*, and cages were changed twice/week. Mice received *ad libitum* standard water until 8–10 weeks of age, at which point cages were randomly assigned water bottles filled exclusively with either a prepared nicotine solution or control solution (described below) available *ad libitum*. Water bottles were replaced with fresh solution twice per week and mice maintained fluid intake exclusively through their designated water preparation for 4 weeks. After 4 weeks of forced nicotine or control water administration, mice went through behavioral testing at 12–14 weeks of age and remained on the designated water solution through the testing period.

#### Sex representation

Adult male and female mice were included in each experimental group and balanced across experimental conditions. Males and females were analyzed separately in our real-time place preference test with optogenetic inhibition (Figs. 2, 3) and no differences were observed by two-way ANOVA multiple-comparisons test between virus expression (EGFP vs NpHR) and sex (male vs female, *p* = 0.80–0.99, data not shown) across each experimental group—nicotine naive (*N* = 7 females, *N* = 9 males), nicotine dependent (*N* = 7 females, *N* = 9 males), and nicotine withdrawn (*N* = 7 females, *N* = 9 males). Importantly, the effect of the virus remained significant within the nicotine withdrawal group independent of animal sex (*p* = 0.03, data not shown), demonstrating a preserved response to optogenetic inhibition during a withdrawal state equivalent across male and female mice. Our experimental design consisted of three conditioning days of exposure to repeated optogenetic inhibition. Therefore, we continued to assess if our place preference response remained significant independent of sex as a variable pooled across the three conditioning days. Again, we observed no sex difference with two-way RM ANOVA with multiple comparisons (*p* = 0.89–0.98, data not shown) with a preserved effect of optogenetic inhibition only in the nicotine-withdrawn group [*p* < 0.0001, data not shown male (*N* = 25) and female (*N* = 19)]. Based on these results the sexes were combined for analysis and subsequent tests did not power those experiments to resolve sex differences. Male and female sexes were incorporated into our experimental design, as females have historically been underrepresented in nicotine literature (Beery, 2018). While our results did not show evidence for sex differences in the manipulation of our circuitry, previous literature shows sex differences play an important role in nicotine addiction and withdrawal (Chellian et al., 2021). This could be partially attributed to IPN being a strong driver of aversion in both male and female mice (Liang et al., 2024). Future dedicated studies to explore the impact of sex in these contexts is important, especially as we gain deeper understanding of the anatomical and physiological properties of these aversive circuits.

### Nicotine administration

Chronic tartrate or nicotine tartrate (200 µg/ml, free base) solution was administered orally, pH 6.5–7.0, for >4 weeks via drinking water with 2% saccharin sweetener added for palatability (Grabus et al., 2005). Three treatment groups were tested in these experiments: nicotine naive, consumed tartrate solution for >4 weeks; nicotine dependent, consumed nicotine tartrate solution for >4 weeks, and nicotine withdrawal, consumed nicotine tartrate for >4 weeks followed by either 24 h removal of nicotine from the drinking water or acute intraperitoneal injection of 2 mg/kg mecamylamine (MEC). Body weight was measured twice per week throughout the nicotine or tartrate drinking period and just prior to any experimental procedure. A threshold of >20% weight loss was set as an exclusion criterion, and no mice were excluded due to excessive weight loss. Forced nicotine drinking at this concentration for 4 weeks generates serum cotinine levels of 150–300 ng ml^−1^ (Fig. 2*B*), which is similar to those observed in heavy smokers (Hukkanen et al., 2005; Arany et al., 2011). This treatment paradigm results in withdrawal behaviors following cessation of nicotine drinking or mecamylamine administration (2 mg/kg; Damaj et al., 2003). While spontaneous withdrawal is more representative of the human condition, we decided our question could best be answered using mecamylamine-induced withdrawal. Many of our experiments require a time-locked induction for a direct comparison before and after withdrawal. Additionally, mecamylamine-induced withdrawal optimizes experimental testing by standardizing the time of withdrawal. With nicotine drinking, the last nicotine exposure is not monitored, which precludes prediction of the timing of maximal withdrawal symptoms, further justifying the use of mecamylamine for withdrawal induction. We realize the importance of spontaneous withdrawal measurements for the translation of studies to humans. We repeated our studies of novelty seeking during nicotine withdrawal combined with optogenetic inhibition of IPN→LDTg GABAergic projections and saw similar results (data not shown), suggesting a similar withdrawal state and circuit recruitment between the two methods as previously shown (Damaj et al., 2003; Zhao-Shea et al., 2013, 2015).

### Drugs and reagents

For nicotine drinking, (−)-nicotine hydrogen tartrate salt (Glentham Life Sciences) or ʟ-(+)-tartaric acid (Sigma-Aldrich) was used in combination with saccharin sodium salt hydrate (Acros Organics, 99+% purity). For precipitated withdrawal, mecamylamine hydrochloride (Abcam) was administered 2 mg/kg intraperitoneally.

### ELISA cotinine assay

In a subset of animals, serum cotinine levels were measured using an ELISA kit from Calbiotech (Lot #: CO096D-100). After experimentation, samples were collected from nicotine-naive, nicotine-dependent, or nicotine-withdrawn mice (24 h without nicotine). Using a 23 ga needle, 150–300 µl cardiac blood was collected from the left atrium. Cardiac blood was transferred to a polypropylene tube and rested at room temperature for 20 min. Blood was centrifuged for 10 min at 3,000 rpm in 4°C. Clear serum was extracted and aliquoted into vials stored at −80°C.

Samples were diluted to 1:3 in PBS and were run in duplicate according to the kit instructions. Absorbances were read on a microplate reader (BioTek Epoch) at 450 nm within 15 min of the Stop Solution. Six standards (0, 5, 10, 25, 50, 100 ng/ml cotinine) were included in duplicate to generate a standard curve (absorbance vs concentration) for quantification of cotinine levels in the serum samples.

### Surgical procedures

Mice underwent surgery 1 week after initiation of nicotine drinking. All surgeries followed aseptic protocol, and tools were sterilized with glass bead sterilizer (FST sterilization tool 18000-45). Stereotaxic surgery (Kopf Instruments) was performed under anesthesia with isoflurane (Covetrus), induced at 2% and maintained at 1%. Body temperature was maintained at 37°C using a homeothermic heating pad (Harvard Apparatus). An ophthalmic ointment was used for eye lubrication. Prior to incision, mice were administered buprenorphine (Hospira, 0.05 mg/kg, s.c.) and bupivacaine (Hospira, 1 mg/kg, s.c.). The surgery site was sterilized with betadine solution prior to surgical incision at the top of the skull.

All viral injections were administered using a Hamilton syringe (1700 series, 33 ga). After full viral infusion, the needle was held in place for 10 min before slow extraction from the brain.

#### Optogenetics

For all nicotine somatic and affective withdrawal behavioral experiments, viral vectors expressing AAV5.Ef1a.DIO.eNpHR3.0-eYFP or AAV5.Ef1a.DIO-eYFP (500 nl, infusion rate 150 nl/min) were injected into IPN (AP: −3.5 mm, ML: 0.9 mm, DV: −4.8 mm from bregma at 10° angle) of Gad2-cre mice with an optical fiber (200 µm) placed 0.2 mm above the LDTg (AP: −5.0 mm, ML: 1.8 mm, DV: −3.3 mm from bregma at 20° angle). Viruses were obtained from Addgene (stock #s: 26966, 27056, respectively). Optical fibers were made using ceramic cannulas (Thorlabs, CFLC230) as described previously (Ung and Arenkiel, 2012).

#### Fiber photometry

AAV9.Syn.GCaMP6s.WPRE.SV40 (500 nl, infusion rate 100 nl/min) was injected into the LDTg (AP: −5.0 mm, ML: 1.8 mm, DV: −3.5 mm from bregma at 20° angle) or AAV9.hSyn.GRAB_DA1h (500 nl, infusion rate 100 nl/min) was injected in the NAc (AP: 1.0 mm, ML: 2.0 mm, DV: −4.3 mm from bregma). An optical fiber (400 µm, MFC_400/430-0.48_5mm_MF1.25_FL, Doric) was placed 0.2 mm above the target nuclei. Viruses were obtained from Addgene (stock #s: 100843, 113050, respectively).

#### Rabies transsynaptic tracing

 AAV8.hSyn.FLEX.TVA.P2A.eFGP.2A.oG (400 nl, 100 nl/min) was injected into the LDTg (AP: −5.0 mm, ML: 1.8 mm, DV: −3.5 mm from bregma at 20° angle) of Gad2-Cre, ChAT-Cre and Vglut2-Cre mice. Subsequently, 14 d after helper virus injection, EnVA G-deleted Rabies-mCherry (200 nl, 100 nl/min) was injected into the LDTg at the same coordinates. Viruses were obtained from Salk Institute for Biological Studies.

To ensure a tight connection, all headcaps contained two skull screws and were secured using dental acrylic (Lang Dental, Jet Denture Repair Powder and Jet Liquid). After surgery, 0.5 ml saline and 5 mg/kg meloxicam (Sigma) were administered subcutaneously. Mice recovered from anesthesia on a heating pad and then were given 3 week recovery and viral expression time before experimentation.

### Brain slice preparation

Brain slices were obtained using a neuroprotective recovery method, as previously described (Wolfman et al., 2018). Mice were rapidly decapitated following anesthesia with isoflurane. Brains were dissected in a solution of ice-cold protective artificial cerebrospinal fluid (aCSF) including the following (in mM): 92 *N*-methyl-d-glucamine, 2.5 KCl, 1.25 NaH_2_PO_4_, 30 NaHCO_3_, 20 HEPES, 25 glucose, 12 N-acetyl cysteine, 2 thiourea, 5 Na-ascorbate, 3 Na-pyruvate, 0.5 CaCl_2_·4H_2_O, and 10 MgSO_4_·7H_2_O, pH adjusted to 7.3–7.4 with HCl and then bubbled continuously with 95% O_2_–5% CO_2_. Then, 250-μm-thick IPN coronal slices were cut with a vibratome (VT100S, Leica) and incubated in a holding chamber at 32–34°C for ≤15–20 min in the same protective aCSF. Slices were then transferred to a holding chamber containing room temperature aCSF including the following (in mM): 119 NaCl, 2.5 KCl, 1.25 NaH_2_PO_4_, 26 NaHCO_3_, 20 HEPES, 12.5 glucose, 5 *N*-acetyl cysteine, 2 thiourea, 5 Na-ascorbate, 3 Na-pyruvate, 2 CaCl_2_·4H_2_O, and 2 MgSO_4_·7H_2_O, bubbled continuously with 95% O_2_–5% CO_2_ and perfused at a rate of 20 ml min^−1^ for at least 30 min before recording.

### Brain slice recording

Recording chambers were superfused (∼2 ml min^−1^) with room temperature aCSF (in mM, 125 NaCl, 25 NaHCO_3_, 20 glucose, 2.5 KCl, 2.5 CaCl_2_, 1 MgCl_2_, 1 NaH_2_PO_4_, at pH 7.4, saturated with 95% O_2_ and 5% CO_2_). Neurons were visualized under infrared illumination using a fixed-stage upright microscope (Axioskop, Zeiss). Data were acquired with a MultiClamp 700A/Axopatch 200B amplifier and pCLAMP 9 software (Molecular Devices). Whole-cell patch-clamp recordings were achieved with microelectrodes (3–6 MΩ) pulled on a Flaming/Brown micropipette puller (model P-97, Sutter Instrument). All electrophysiology experiments were performed on neurons in the IPN that expressed NpHR (verified by EYFP fluorescence). Recording electrodes were filled with potassium gluconate internal solution (in mM): 154 K-gluconate, 1 KCl, 1 EGTA, 10 HEPES, 10 glucose, 5 ATP, 0.1 GTP, pH 7.4 with KOH. To activate light-sensitive NpHR, light was delivered through the objective at maximal power (>40 mW; 532 nm). Light evoked inhibitory currents were recorded in voltage-clamp mode with 3 ms stimulation. Light evoked membrane hyperpolarization was recorded in current-clamp mode with prolonged 10 s stimulation.

### Histology

Animals were anesthetized with isoflurane and transcardially perfused with 4% paraformaldehyde. Brains were kept in paraformaldehyde for >24 h and then transferred to 30% sucrose in phosphate-buffered saline (PBS) for >24 h. Brains were frozen in embedding medium (OCT Compound, Fisher). Injection sites and optical fiber placements were confirmed in all animals by taking coronal sections (60 µm) of perfused tissue using a cryostat (Leica CS3050 S). Animals with incorrect placement of either fiber optics (>200 µm from target) or viral injections were excluded from analysis. Viral injection criteria for exclusion were >10% expression outside of nucleus for optogenetic experiments and >0% starter cells within 1 mm of tissue outside of LDTg at injection site for rabies transsynaptic tracing.

For optogenetic experiments, in a subset of sections (20 µm), immunohistochemistry (IHC) was used to confirm cre-dependent expression of NpHR using rabbit α-GAD65 + 67 (1:1,000, Abcam 11070) with Alexa donkey α-rabbit 647 (1:1,000, Thermo Fisher A-31573).

For rabies transsynaptic tracing, sections were taken at 40 µm and downsampled to every other section throughout the entire brain. IHC was performed to boost helper virus expression (GFP), rabies virus expression (mCherry), and stain for GAD67 using the following primary antibodies: rabbit α-GFP (1:2000, Abcam ab290), chicken α-mCherry (1:1,000, Origene TA150127), and goat α-Gad67 (1:1,000, R&D AF2086). Respectively, secondary antibodies used were as follows: Alexa donkey α-rabbit 488 (1:2000, Thermo Fisher A-21206), Alexa donkey α-chicken 594 (1:1,000, Thermo Fisher A-78951), and Alexa donkey α-goat 647 (1:1,000, Thermo Fisher A-21447).

For all IHC, sections were washed in PBS in between the following steps: incubation in blocking solution (1% BSA, 10% NDS, and 0.1% Triton X-100 in PBS) for 2 h at room temperature, in primary antibodies for 24 h in PBS+ blocking solution at 4°C, and in secondary antibodies for 2 h at room temperature in PBS. Sections were mounted on slides (Fisherbrand, Superfrost Plus) with DAPI Fluoromount-G (Southern Biotech) and coverslipped (Fisher Scientific). Images were taken on a 3i Marianas Spinning Disk Confocal or Olympus VS200 Slideview Research Slide Scanner.

Cell detection and fluorescent cell counts were done on QuPath software. Cell detection was done using DAPI nucleus staining, and cytoplasm estimates were determined given parameters for area (max/min), intensity threshold, and cell expansion. A Gaussian filter was applied for noise reduction. For positive cell detection with fluorescent staining, a single measurement classifier was used to detect positive nuclei or cytoplasm containing intensity levels above target threshold. For colocalization, classifiers were applied sequentially to region of interest.

### Behavior

Behavioral experiments occurred after 4 weeks of chronic tartrate (nicotine naive) or nicotine drinking (nicotine dependent), which overlapped with viral expression and recovery time from surgery.

#### Optogenetics

For optogenetic behavioral experiments, implanted cannulas were hooked to an optical fiber cable with an inbuilt rotary joint (RJPFL4 outer diameter 1.25 mm, core diameters 400 μm, Thorlabs). To activate NpHR opsin, we used a 532 nm laser (DPSS) to deliver constant light. Mouse behaviors were recorded using a camera (Basler) connected to EthoVision XT-16 software (Noldus). Using a Master-9 Pulse Stimulator (A.M.P.I), the light was triggered under the appropriate experimental conditions. In all optogenetic experiments, the light at the tip of the cannula was adjusted to ∼5 mW (10 mW/mm^2^) peak power using a power meter (Thorlabs, PM20A). All behavioral apparatuses were cleaned with 70% ethanol after each session. Experimenters were blinded to NpHR or eYFP expression. NpHR function was tested using ex vivo electrophysiology.

#### Real-time place preference

Prior to testing, mice were handled and habituated to intraperitoneal injections for ≥3 d. A two-chambered box (each chamber 25 cm × 25 cm × 25 cm) was used to assess place preference in a real-time assay (20 min/d). Each chamber of the boxes has different patterned walls (vertical vs horizontal, black and white stripes) and different textured floors (ribbed lines vs patchwork). Mice were connected to optical fiber cord for all behavioral test days.

Experimental groups included (1) nicotine-naive, (2) nicotine-dependent, and (3) mecamylamine (MEC)-precipitated nicotine withdrawal. Within these three groups, mice underwent either control (eYFP) or halorhodopsin (NpHR) viral surgeries. Mice received an intraperitoneal injection immediately preceding real-time test. Day 1: All mice received a saline injection (SAL) intraperitoneally. Days 2–4: Nicotine-naive and nicotine-withdrawn mice received an injection of 2 mg/kg MEC intraperitoneally. Nicotine-dependent mice received a SAL injection intraperitoneally. Day 5: Nicotine-naive and nicotine-withdrawn mice received either SAL or 2 mg/kg MEC intraperitoneally. Nicotine-dependent mice received a SAL injection intraperitoneally.

Real-time place preference test timeline: Day 1: This was a pretest day to assess baseline preference with the laser off. All mice with a >70% preference for either chamber were excluded from the experiment. Days 2–4: In an unbiased design, mice received constant 532 nm light delivered through a DPSS laser (SLOC) and gated to turn on while in one of the two chambers controlled using EthoVision 11 software (Noldus) and a Master-9 pulse stimulator. The light-paired chamber was kept consistent within animals across the 3 d of real-time place preference and counterbalanced across animals. Light power output was tested using a digital power meter (Thorlabs) and was checked before and after each experimental animal. Output during light stimulation was estimated to be 4–6 mW/mm^2^ at the targeted tissue 0.2 mm from the fiber tip. Day 5: Posttest day to assess conditioning after 3 d of light-paired inhibition with a specific chamber.

#### Open field and novel object test

In a separate set of animals, mice were split into nicotine-naive and nicotine-dependent groups with either eYFP or NpHR virus expressed. Prior to testing mice were handled and habituated to intraperitoneal injections for ≥3 d. A large box (each chamber 20 cm × 43 cm × 43 cm) was used to assess locomotion in a 20 min open field test. Immediately preceding open field test, mice were injected with 2 mg/kg MEC and placed immediately in the center of the arena. Constant 532 nm light delivery occurred during the entirety of the test. After 10 min, a novel object was presented into the center of the chamber. Object interaction was defined when the mouse approached the object with its nose within 2 cm or less.

#### Somatic withdrawal signs and social odor preference

In a separate set of animals, mice were grouped by nicotine naive and nicotine dependent with either eYFP or NpHR virus expressed. Prior to testing mice were handled and habituated to intraperitoneal injections for ≥3 d. One hour before experimentation, mice habituated to two cotton swabs affixed to opposite sides of the home cage. After injection of 2 mg/kg MEC, mice were placed in their home cage with one swab dipped in water and one swab dipped in the urine of a mouse of the opposite sex. During the 3 min test, mice were allowed to freely explore the home cage and sniff the two swabs with constant 532 nm light delivery. Immediately after the social odor preference assay, mice were moved to a large plexiglass box with an angled mirror underneath to assess somatic signs for 10 min. Time spent sniffing and somatic signs were analyzed through video analysis by a scorer blinded to experimental conditions.

#### Behavioral analyses

All exposures to the apparatus were recorded with a video camera and analyzed using EthoVision 11 (Noldus). For the real-time place preference test, a preference score (%time spent in light-paired chamber − %time spent in non-light-paired chamber) and a change in preference (preference score on experimental day − preference score on pretest day) were calculated. Position traces were automated by EthoVision software using center-point detection of the mouse and dynamic subtraction from the background. For the open field test, time spent and number of visits to the center zone (21 cm × 21 cm) were determined by the software. For novel object test, time spent and number of object interactions was measured by scorer blinded to animal group. For somatic signs, a blinded scorer counted the following signs and their durations that were visible by video analysis: grooming, paw licking, straub tail, shaking, rearing, backing, retropulsion, head nodding, abdominal gasps, and jumping. Some signs reported in previous studies were excluded due to inability to distinguish in the videos, including yawning, chewing, facial fasciculations, cage scratching, ptosis, and piloerection. Individual signs were included only when >3 occurrences were observed. Blinded scorer was trained on wild-type behavioral data, previously verified by three separate scorers. For social odor test, a blinded scorer and a live unblinded scorer counted sniffing time. If discrepancy was >0.5 s the blinded score was used, otherwise, an average was taken.

### Fiber photometry

Mice were habituated to handling, vehicle injection, and recording apparatus for ≥3 d prior to experimentation. Photometry setup included optical components from Doric Lenses controlled by a real-time processor from Tucker-Davis Technologies (TDT; RZ5P). Photometry rig included lock-in amplified and processor to drive and demodulate signals, allowing for monitoring of calcium-dependent and calcium-independent signals. TDT Synapse software was used for data acquisition, timestamp events with TTL loggers, and LED control. Two excitation wavelengths of 465 nm (calcium dependent) and 405 nm (isosbestic control) LEDs were modulated at 210 and 330 Hz, respectively. The emitted fluorescence was recorded using a photodetector (Newport Model 2151) and sent to a data acquisition device via mini cube port to isolate individual signals. LED currents were adjusted to return a comparable baseline voltage in the calcium-dependent and isosbestic channels, and this level was maintained within animals across experiments. Since the signal was sampled at 1,017.3 Hz, we ensured that no signal was modulated at a frequency greater than half the sampling frequency to prevent aliasing errors. Signal was collected with 8 Hz low-pass frequency filter. Behavioral timestamps were fed into the real-time processor as TTL signals for alignment with the neural data or timestamped from a camera log.

In LDTg (GCaMP6) and NAc (GRAB-DA) recordings, mice were habituated to the recording room for 1 h. Mice were connected to the fiber-optic cable and allowed to explore the apparatus for 5 min. After recording started, baseline signal was recorded for at least 5 min in freely moving mice. Mice were injected with SAL or 2 mg/kg MEC intraperitoneally and recorded for 10 min. Response to novel object (10 min test) and baseline occurred on separate days postinjection of SAL or 2 mg/kg MEC, as described above. Different novel objects were used for SAL and MEC recordings for within-animal comparisons.

To validate the signals from GRAB-DA, a modified D2 DA receptor, some mice were injected with 1 mg/kg *S*-(−)-eticlopride hydrochloride (Sigma-Aldrich; i.p. injection), a D2R antagonist. Fluorescent transients were reduced compared with a 10 min baseline period.

For analysis, the signal from the 405 and 465 nm channels were extracted using MATLAB software according to published protocols (Bruno et al., 2021; Sherathiya et al., 2021). The photo-bleaching of GCaMP over long sessions was removed using a double exponential fit to the entire dataset. We subtracted the calcium-independent signal from the calcium-dependent signal to reduce movement or hemodynamic artifacts. To that end, a smoothed 405 nm isosbestic signal was fitted to the 465 nm signal using linear regression to obtain fitting coefficients. The fitted control was used to calculate the change in fluorescence (Δ*F*/*F*) or *z*-score. Using the fitting coefficients, the “fit 405 nm” signal was calculated, subtracted, and divided from the 465 nm signal to obtain a Δ*F*/*F* ([*F*_465_ − *F*_fit405_] / *F*_fit405_). A robust *z*-score based on the median Δ*F*/*F* was calculated for the concatenated Δ*F*/*F* data for all sessions for individual mice to facilitate comparison across mice and sessions. This robust *z*-score was calculated by first removing the high amplitude events (>2× median absolute deviation) and identifying the median of the filtered trace. This median was subsequently used to normalize the Δ*F*/*F*. Parameters including number of peaks, peak amplitude, peak half-width, and AUC were measured from *z*-score. The frequency and amplitude of transients were calculated using the *findpeaks* command in MATLAB with a prominence value of 2.9× standard deviation of the data. The area under the curve was calculated using the trapezoidal method for integrals (*trapz*). For novel object data, a time window of 500 ms was used to measure AUC for the transient activity time-locked to novel object interaction. This method limited the confounding effects of continued transient events unrelated to novel object interaction. However, analysis of the AUC values for the full dataset gave similar behaviorally relevant transients, statistics, and interpretation.

### Randomization and blinding

Cages were selected arbitrarily to receive viral injections (eYFP vs NpHR) and drinking water (tartrate vs nicotine). Mice were randomly assigned to behavioral boxes and light pairing was randomized across behavioral boxes, taking care to alternate orientation of chamber (vertical vs horizontal stripes) within the behavior testing room. Behavioral tests were performed by investigators with knowledge of the tartrate versus nicotine experimental groups, but they were blinded to eYFP or NpHR expression. Where possible, behavioral experiments were controlled by computer systems, and data were collected and analyzed in an automated and unbiased way. For somatic signs, novel object interaction, and social odor tests, the behavior was scored by an experimenter blinded to drinking water and viral expression groups. The experimenter was trained on wild-type data that was previously standardized by three other blinded observers. Histological validation of viral expression and fiber-optic placement always took place prior to analysis of behavioral data. Experimenters were not blinded to the groups during this verification step but were blinded to the actual observed behavior of individuals and groups.

### Data analysis

Data were tested for normal distribution using Shapiro–Wilk or D’Agostino and Pearson (repeated measures) tests. For data that conformed to normal distribution, Student's *t* tests (paired and unpaired); one-way, two-way, or three-way ANOVA (normal or repeated measures) tests; and mixed-effects analyses were used to determine statistical differences using GraphPad Prism 9 (GraphPad software). Tukey's HSD or Holm–Sidak's post hoc analysis was applied when an ANOVA or mixed-effects analysis showed a significant main effect or significant interaction for multiple comparisons. For non-normal distribution, Kruskal–Wallis or a Mann–Whitney test was used followed by Dunn's multiple-comparisons test (as needed) when analysis showed a significant main effect. A Welch's correction was done for data where equal SD could not be assumed. Statistical significance (*p* value) is listed in the figure legends. All data are presented as mean ± SEM.

## Results

### Inhibition of GABAergic IPN→LDTg inputs generates preference during nicotine withdrawal

**Figure 1.**
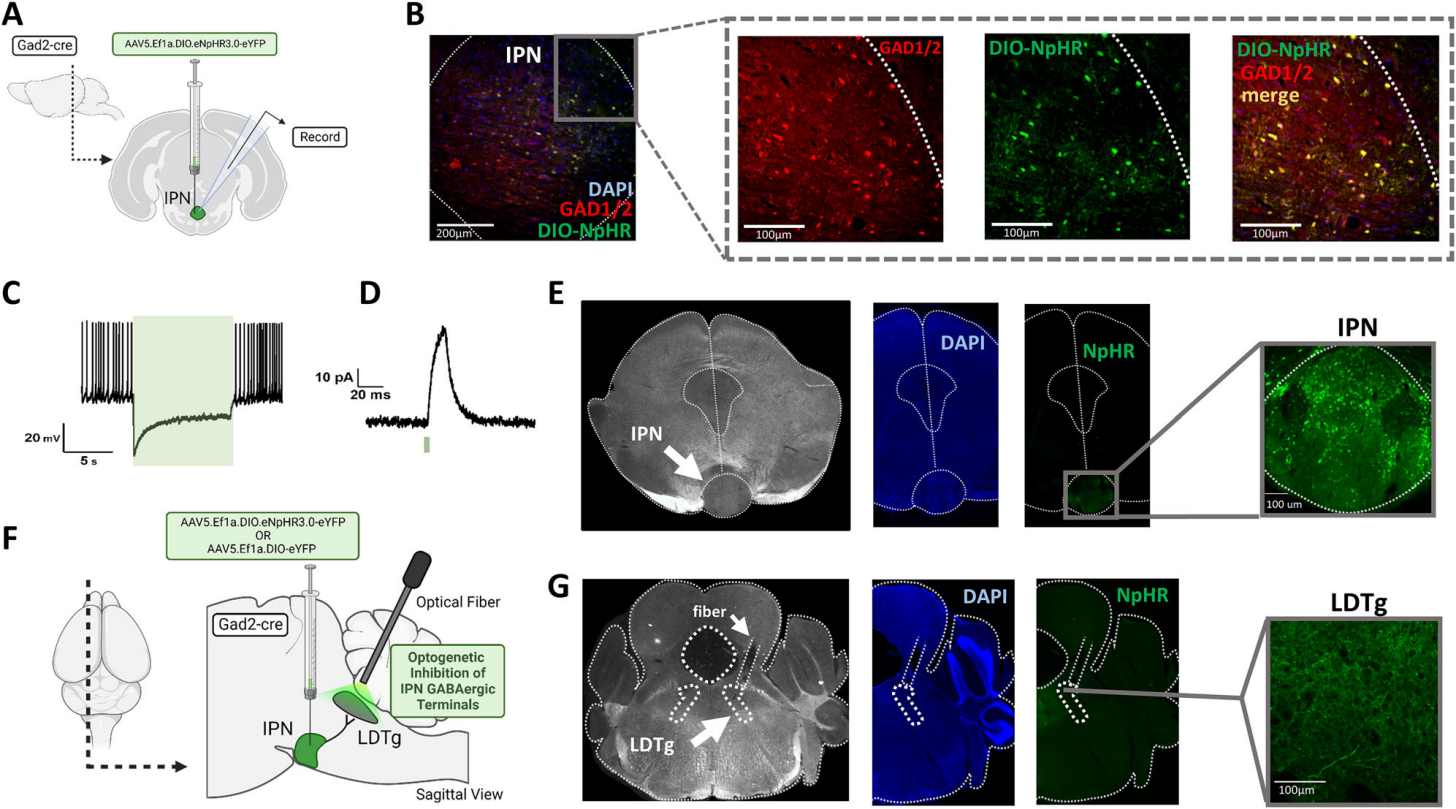
Optogenetic inhibition of IPN GABAergic terminals in LDTg. ***A***, Surgery and electrophysiology schematic illustrating cre-mediated expression of NpHR in GAD2-cre mice. ***B***, Viral vector cre-mediated expression of NpHR (green) overlapping (yellow) with immunohistochemistry staining of GAD1/2 (red) in the IPN of GAD2-cre mice. ***C***, Light evoked hyperpolarization of membrane potential in ex vivo recording from an IPN neuron expressing eYFP-tagged NpHR. ***D***, Light evoked inhibitory outward current in ex vivo recording from eYFP-labeled IPN neuron expressing NpHR (*Vm* = −70 mV). ***E***, Histologic confirmation of viral injection and eYFP-NpHR localization within the IPN. ***F***, Surgery schematic illustrating cre-mediated expression of NpHR or eYFP in IPN of GAD2-cre mice and fiber-optic placement above the LDTg for light-evoked inhibition of terminals. ***G***, Histologic confirmation of fiber-optic above the LDTg with eYFP-NpHR fluorescence in IPN neuron axon terminals.

**Figure 2.**
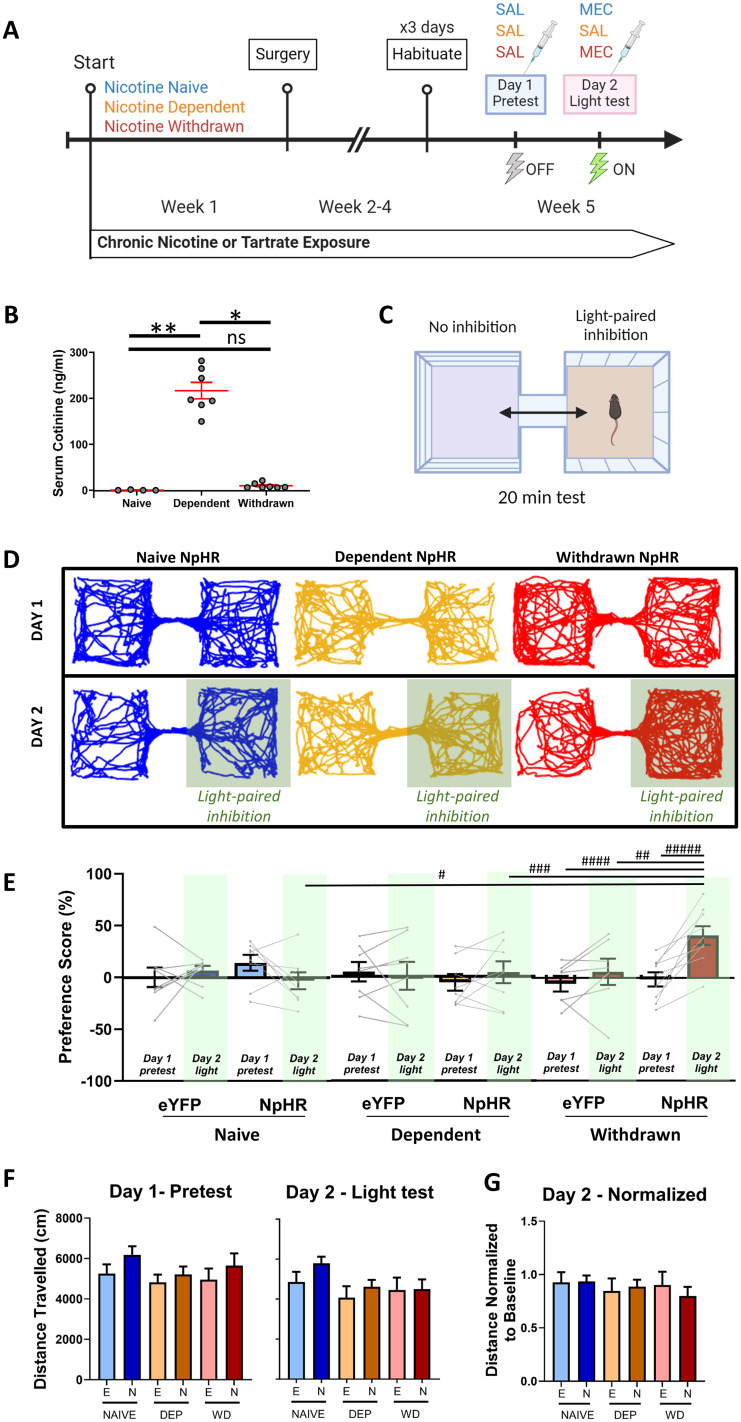
Inhibition of IPN→LDTg GABAergic terminals during nicotine withdrawal results in place preference. ***A***, Timeline of nicotine drinking, surgery, and behavior testing. Nicotine withdrawal is precipitated with a 2 mg/kg mecamylamine (MEC) injection after 4 weeks of forced nicotine drinking. ***B***, Serum cotinine levels were measured by ELISA in mice that drank tartrate water (nicotine naive, *N* = 4), nicotine water for 4 weeks (nicotine dependent, *N* = 7; 200 µg/ml, free base), and 24 h after removal of nicotine from the drinking water at the end of the 4 weeks (nicotine withdrawn, *N* = 7). Four weeks of nicotine drinking resulted in elevated serum cotinine levels, which return to baseline 24 h after removal of nicotine (Kruskal–Wallis Dunn's multiple comparisons, *H* = 14.92, *p = 0.0419, ***p = 0.0005). ***C***, Real-time place preference behavioral apparatus with different patterned walls and different textured floors for conditioning. Light-paired chambers were designated using an unbiased, randomized design. ***D***, Positional traces representing the first 10-min of testing on Day 1 (pretest) and Day 2 (light inhibition; green box, paired side) for NpHR-mediated IPN→LDTg GABAergic terminal inhibition in nicotine-naive (blue), nicotine-dependent (orange), and nicotine-withdrawn (red) mice. ***E***, Preference score (preference score = % time spent in light-paired chamber − % time spent in non-paired chamber) for the light-paired versus non-paired chamber for EYFP or NpHR expressing mice under nicotine-naive (eYFP, *N* = 8; NpHR, *N* = 8), nicotine-dependent (eYFP, *N* = 8; NpHR, *N* = 8), or nicotine-withdrawn (eYFP, *N* = 7; NpHR, *N* = 9) conditions. Photoinhibition of IPN GABAergic terminals in LDTg is preferred in MEC-precipitated nicotine withdrawal state (three-way RM ANOVA, Holm–Sidak's multiple comparisons, SS = 3,120, df = 2, *F* = 4.010, ^#^*p* = 0.0401, ^##^*p* = 0.0332, ^###^*p* = 0.0191, ^####^*p* = 0.0031, ^#####^*p* = 0.0026). ***F***, Distance traveled over 20 min during Day 1 pretest (no light inhibition) and Day 2 (with light inhibition), showing no difference in locomotion between EYFP (E) and NpHR (N) expression or nicotine treatment history. ***G***, Distance traveled on Day 2 testing normalized to the baseline (Day 1) average for each group (two-way RM ANOVA, Tukey's HSD multiple comparisons, no significant interaction, SS = 0.04470, df = 2, *F* = 0.3235, *p* = 0.7254). Groups: NAIVE, nicotine naive; DEP, nicotine dependent; WD, nicotine withdrawal.

Previous data implicate an important role of IPN in nicotine withdrawal (McLaughlin et al., 2015), but IPN cell type(s) and downstream target areas that mediate withdrawal symptoms have not been explored. Based on our previous studies demonstrating that IPN projections to the LDTg mediate the aversive effects of high-dose nicotine (Wolfman et al., 2018), we hypothesized that this projection could contribute to the aversive experience of nicotine withdrawal. To test this idea, we expressed either the inhibitory opsin NpHR or eYFP selectively within GABAergic neurons in IPN using stereotaxic injection of viruses in Gad2-Cre mice (Fig. 1*A*,*B*,*E*,*F*), with a fiber-optic implant to deliver light to inhibit GABAergic IPN terminals in the LDTg (Fig. 1*F*,*G*). Verification of opsin function was achieved using brain slice electrophysiology recordings in the IPN, showing light stimulated hyperpolarization and inhibitory currents (Fig. 1*C*,*D*). Nicotine-dependent and nicotine-naive control mice were generated by replacing home cage drinking water with either nicotine tartrate (200 µg/ml nicotine, free base) or tartrate alone (molar equivalent), sweetened with 2% saccharin for palatability, and only one of these solutions was the sole water source in the home cages for 4 weeks (Fig. 2*A*). This nicotine exposure paradigm was sufficient to raise serum cotinine levels to concentrations observed in human smokers (Pekonen et al., 1993; Grabus et al., 2005; Hukkanen et al., 2005), and then, 24 h after removal of nicotine from the drinking water, cotinine dropped to background levels, consistent with elimination of nicotine and its metabolites from the systemic circulation (Fig. 2*B*).

**Figure 3.**
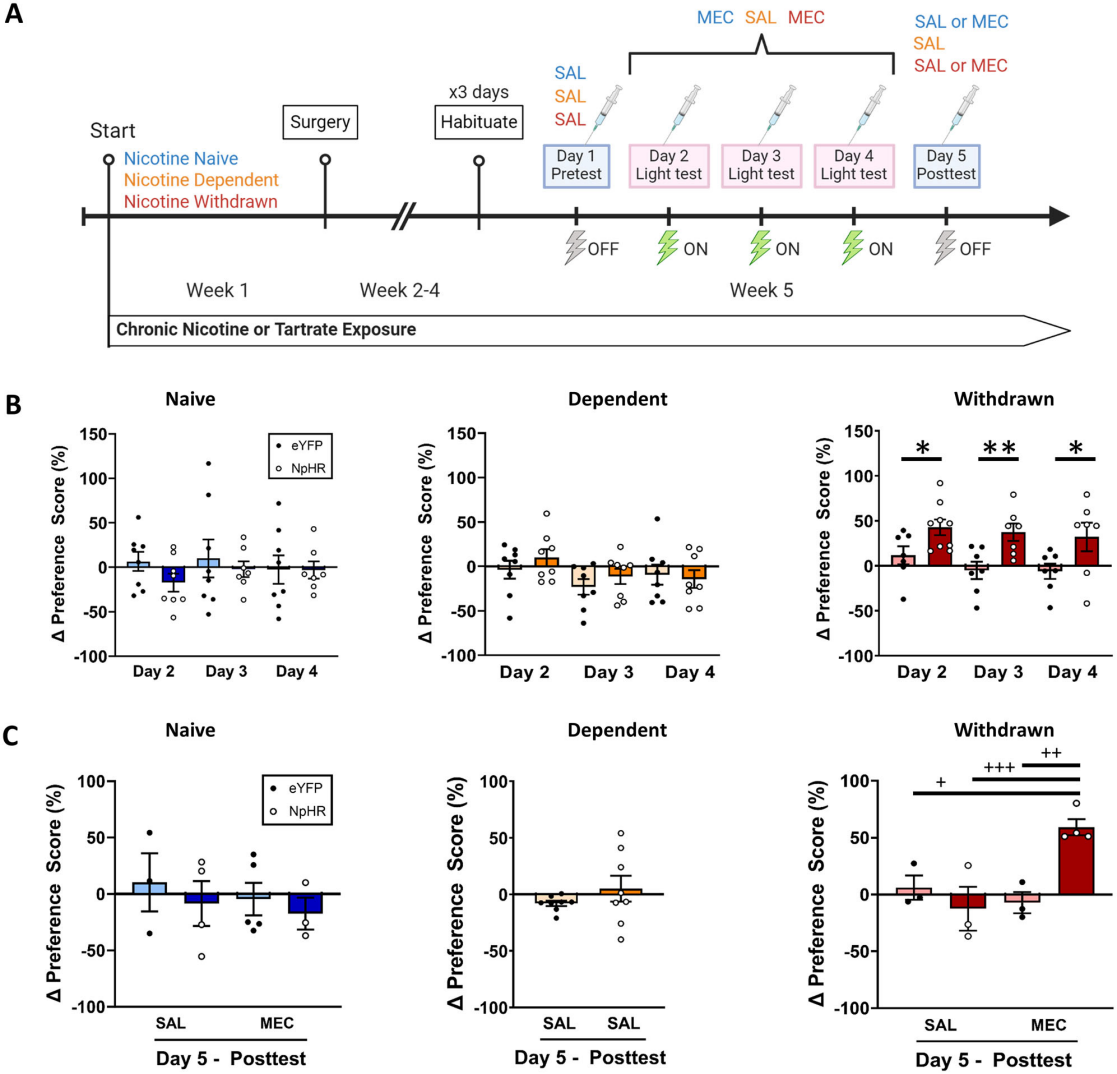
Context-dependent conditioning for photoinhibition of IPN→LDTg GABAergic projections in nicotine withdrawal. ***A***, Timeline of nicotine drinking, surgery, and behavior testing. Behavior included pretest day, 3 d of conditioning to light-paired inhibition of IPN→LDTg terminals, and posttest day. On posttest day mice were split into two groups, receiving either saline (SAL) or 2 mg/kg MEC injections to assess context-dependent effect of conditioning. ***B***, Preference scores were determined (% time spent in light-paired chamber − % time spent in non-light-paired chamber) and then the change (Δ) in preference scores (% preference score test day − % preference score pretest day) were calculated for all three conditioning days in nicotine-naive (blue; eYFP, *N* = 8; NpHR, *N* = 8), nicotine-dependent (orange; eYFP, *N* = 8, NpHR, *N* = 8), and nicotine-withdrawn (red; eYFP, *N* = 7, NpHR, *N* = 9) groups. An increase in preference for light evoked optogenetic inhibition of IPN→LDTg GABAergic terminals was observed in nicotine-withdrawn mice that was maintained across 3 d of testing (RM mixed-effects model, Holm–Sidak's multiple comparisons, *W* = 0.8748–0.9497, *p = 0.0384, **p = 0.0021), while no change in preference was observed between EYFP and NpHR in the nicotine-naive and nicotine-dependent groups. ***C***, Following 3 d of conditioning, Δ preference score on posttest day revealed conditioned place preference for the light-paired side only in the nicotine withdrawal group (red) that were concurrently in the withdrawn state (MEC injection; *N* = 4). Mice in the same group that were not experiencing withdrawal (SAL injection; *N* = 3) did not show a side preference during the testing period (two-way ANOVA, Holm–Sidak's multiple comparisons, SS = 5,780, df = 1, *F* = 19.02, ^+^*p* = 0.0126, ^++^*p* = 0.0038, ^+++^*p* = 0.0026). There was no change in posttest Δ preference score across nicotine-naive and nicotine-dependent conditions.

Mice were group housed and randomly assigned to the three experimental groups: nicotine naive (tartrate control cages), nicotine dependent [nicotine drinking cages + saline (SAL) injection], or nicotine withdrawal [nicotine drinking + mecamylamine (MEC) injection; Fig. 2*A*]. We used a 2 mg/kg dose of MEC, which is known to precipitate withdrawal symptoms in mice chronically exposed to nicotine (Damaj et al., 2003). In our experiments, nicotine withdrawal affective behavior was tested using a real-time place preference test (Fig. 2*A*,*C*,*D*). The real-time behavior boxes had distinct patterned walls and textured floors (Fig. 2*C*). Fiber-optic delivery of light to inhibit GABAergic IPN axon terminals in the LDTg resulted in place preference during nicotine withdrawal, but not in nicotine-naive or nicotine-dependent mice (Fig. 2*D*,*E*). Testing the same cohort of mice over 3 d revealed similar preference effects, where the nicotine withdrawal group that experienced NpHR inhibition maintained a preference for the light-paired chamber (Fig. 3*A*,*B*). This repeated testing over 3 d also generated a conditioning effect, as light inhibition of IPN terminals in LDTg was paired with a specific chamber in an unbiased manner (Steidl et al., 2017). After the three conditioning days, a posttest assessment revealed conditioned preference to the previously light-paired chambers (Fig. 3*A*,*C*). Interestingly, preference for the chamber conditioned with optogenetic inhibition occurred only if the mice were experiencing nicotine withdrawal on the posttest day. Mice in the nicotine-dependent state, regardless of their past experiences, showed no preference for the previously light-paired chamber (Fig. 3*C*). Importantly, inhibition of GABAergic IPN→LDTg terminals did not alter locomotion under our experimental conditions (Fig. 2*F*,*G*).

### Inhibition of IPN→LDTg GABAergic terminals reduces somatic signs of nicotine withdrawal

**Figure 4.**
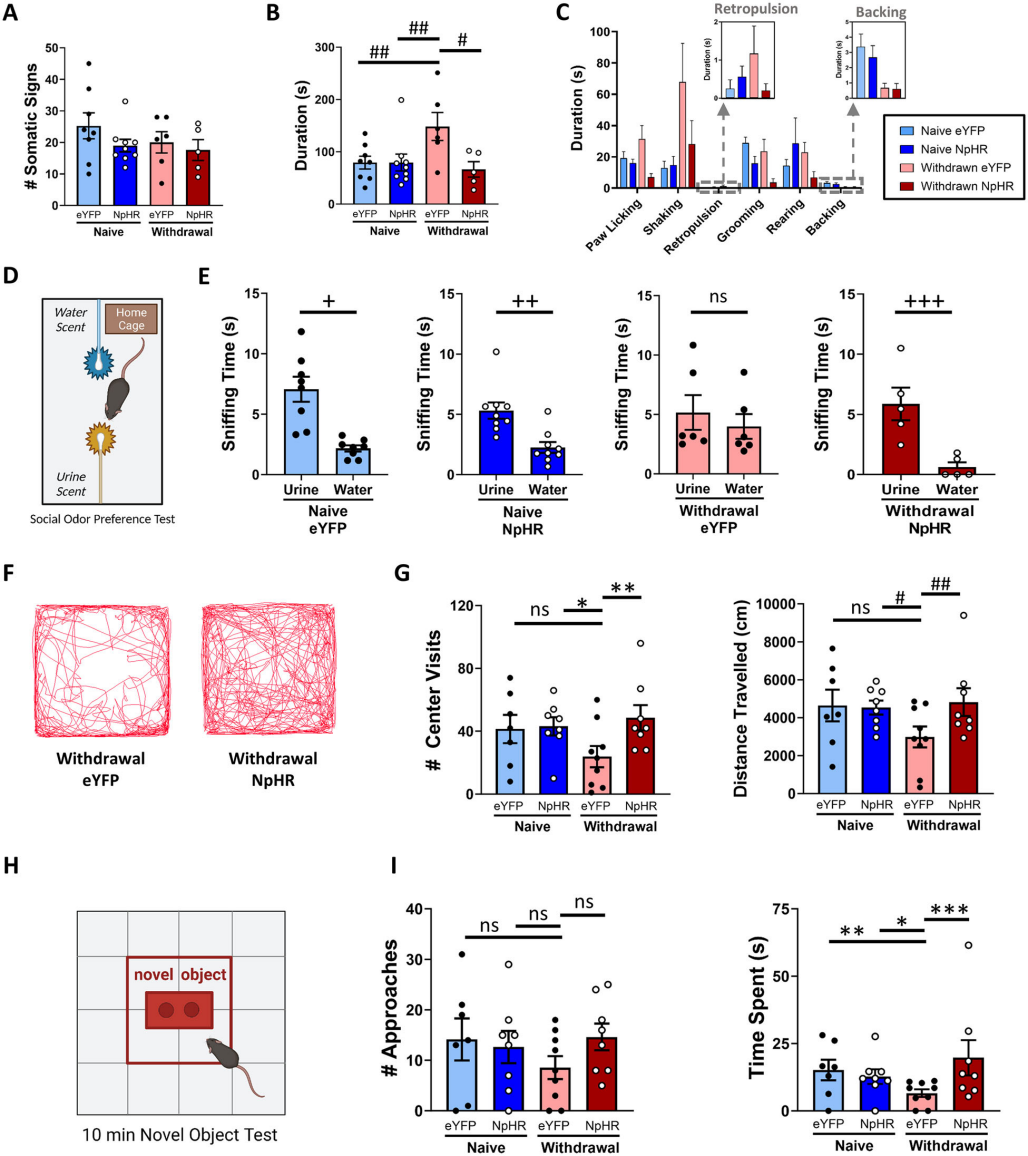
Silencing GABAergic IPN projections to LDTg decreases the severity of nicotine withdrawal behaviors. ***A***, Somatic signs were scored by number and duration during a 10 min test after injection of 2 mg/kg MEC (Withdrawal). Total number of pooled somatic signs (grooming, paw licking, straub tail, shaking, rearing, backing, retropulsion, head nodding, abdominal gasps, jumping) did not change across nicotine exposure (nicotine naive, blue; nicotine exposed, red) or optogenetic inhibition (EGFP, light colors; NpHR, dark colors). Naive eYFP, *N* = 8; Naive NpHR, *N* = 9; Withdrawal eYFP, *N* = 6; Withdrawal NpHR, *N* = 5. ***B***, Total duration of somatic signs was increased during nicotine withdrawal, and optogenetic inhibition of IPN→LDTg GABAergic terminals reversed that effect (two-way ANOVA, Holm–Sidak's multiple comparisons, SS = 11,156, df = 1, *F* = 5.076, ^#^*p* = 0.0477, ^##^*p* = 0.0500). ***C***, Duration of individual somatic signs with >3 occurrences in each experimental group. ***D***, Social odor preference test schematic. Three minute tests occurred in home cage with one swab dipped in water and one swab dipped in the urine of a mouse of the opposite sex. ***E***, Time spent sniffing of the water- and urine-dipped swab for each group. Preference for urine swab (social scent) is lost during nicotine withdrawal, but optogenetic inhibition of IPN→LDTg GABAergic projections during nicotine withdrawal preserves social odor preference (three-way RM ANOVA, Holm–Sidak's multiple comparisons, SS = 29.09, df = 1, ^+^*p* = 0.0308, ^++^*p* = 0.0138, ^+++^*p* = 0.0065). Naive eYFP, *N* = 8; Naive NpHR, *N* = 9; Withdrawal eYFP; *N* = 6, Withdrawal NpHR, *N* = 5. ***F***, Representative position traces of open field test (first 10 min) during nicotine withdrawal showing more center exploration following optogenetic inhibition of IPN→LDTg GABAergic terminals (NpHR) relative to control mouse (eYFP). ***G***, Number of center visits and distance traveled in open field test are plotted for each group. Nicotine withdrawal reduced both parameters (eYFP) but not during optogenetic inhibition (NpHR) of IPN→LDTg GABAergic terminals (# center visits: two-way ANOVA, Holm–Sidak's multiple comparisons, SS = 1,053, df = 1, *F* = 2.417, *p = 0.0499, **p = 0.0318; distance traveled: SS = 7509801, df = 1, *F* = 2.367, ^#^*p* = 0.0380, ^##^*p* = 0.0302). Naive eYFP, *N* = 7; Naïve NpHR, *N* = 8; Withdrawal eYFP, *N* = 9; Withdrawal NpHR, *N* = 8. ***H***, Schematic of novel object test. ***I***, The number of approaches to a novel object does not change with nicotine exposure, withdrawal, or optogenetic inhibition. The time spent sniffing a novel object is decreased during nicotine withdrawal (eYFP) relative to naive mice, but optogenetic inhibition (NpHR) of IPN→–LDTg GABAergic connections increased time spent to naive levels (Kruskal–Wallis, Dunn's multiple comparisons, *H* = 7.987, *p = 0.0463, **p = 0.0220, ***p = 0.0156). Naive eYFP, *N* = 7; Naive NpHR, *N* = 8; Withdrawal eYFP, *N* = 9; Withdrawal NpHR, *N* = 8.

To explore the impact of IPN→LDTg projections in the expression of somatic signs of nicotine withdrawal, mice were randomly divided into nicotine and tartrate drinking cages for 4 weeks. Using MEC to induce nicotine withdrawal, we monitored somatic withdrawal signs with and without light-mediated inhibition of GABAergic IPN→LDTg projections. There is notable inconsistency in the types of somatic signs reported across the literature, with different labs scoring different behaviors (Chellian et al., 2021; Nishitani et al., 2021). For our experiments, we monitored all the somatic signs reported in the literature, pooling those observations in the analyses. In control mice, we did not observe differences in the number of somatic signs displayed between tartrate and nicotine-treated groups (Fig. 4*A*), in contrast to previous reports (Salas et al., 2004; Chellian et al., 2021). However, we did observe an increase in the duration of time spent performing somatic behaviors in mice that received MEC to induce nicotine withdrawal, and this effect was reversed by NpHR-mediated inhibition of the IPN→LDTg projections (Fig. 4*B*). Longer bouts of performing a specific behavior during nicotine withdrawal contributes to the lack of elevation in overall somatic sign number. Of the observable somatic behaviors, shaking and paw licking had the longest durations of the somatic signs observed during nicotine withdrawal (Fig. 4*C*). These data support a role for IPN→LDTg GABAergic projections in the expression of somatic signs during nicotine withdrawal.

### IPN→LDTg projections mediate nicotine withdrawal changes in behavioral response to novel social stimuli

Anhedonia is the reduced ability to experience pleasure (Gorwood, 2008) and is another symptom of nicotine withdrawal (Johnson et al., 2008). The social odor preference test has been described as measure of reward-seeking in mice through behavioral interaction, vocalization, and NAc DA release (Malkesman et al., 2010) and has been used as a measure of hedonic response in the literature (Tadmor et al., 2017). As described previously, mice were randomly divided into nicotine-naive and nicotine-dependent groups. For testing, mice were presented with two swabs in the home cage dipped in either urine of a mouse of the opposite sex or water, separated by the length of the cage (Fig. 4*D*). Urine odor is strongly motivating in mice and sniffing of urine is associated with increases in DA levels within the NAc (Malkesman et al., 2010). Urine is sniffed for longer duration than water or extracts of other odors such as vanilla or almond (Malkesman et al., 2010; Tadmor et al., 2017). We observed greater sniffing time of the urine over water or dry swab, as previously reported (Fig. 4*E*). We observed a loss of preference for the social odor during nicotine withdrawal. However, this change was not observed in mice undergoing nicotine withdrawal that received optogenetic inhibition of the GABAergic IPN→LDTg terminals (Fig. 4*E*). These data suggest that IPN projections to LDTg contribute to changes in social odor preference during nicotine withdrawal.

### IPN→LDTg projections mediate nicotine withdrawal behavioral response to novelty

In addition to somatic signs, IPN has been shown to mediate the affective symptoms of nicotine withdrawal (Fowler et al., 2013; Zhao-Shea et al., 2015; Pang et al., 2016). To test if the IPN connections to LDTg mediate affective behaviors, we tested our optogenetic inhibition of IPN terminals in LDTg in open field and novel object exploration. Nicotine-naive, nicotine-dependent, and nicotine-withdrawn mice displayed distinct levels of distance traveled and numbers of visits to the center zone in the open field test (Fig. 4*F*,*G*), consistent with previous reports (Chellian et al., 2021). Time spent in the center zone was not different between groups (data not shown). After optogenetic inhibition of IPN→LDTg GABAergic terminals, nicotine-withdrawn mice increased their distance traveled and the number of visits to the center zone to levels consistent with nicotine-naive controls (Fig. 4*F*,*G*).

Presentation of a novel object within the open field arena elicited approach behavior in all groups (Fig. 4*H*). We did not see any difference in the number of novel object approaches between groups, but the nicotine-withdrawn mice spent less time interacting with the novel object (Fig. 4*I*). Optogenetic inhibition of GABAergic IPN→LDTg projections reversed the effects of nicotine withdrawal on novel object interactions (Fig. 4*I*). Together, these results suggest that IPN→LDTg inputs contribute to nicotine withdrawal effects on affective behavior. The findings are consistent with a role for this neural connection in anxiety-associated behaviors.

### LDTg neural activity during novel object interaction is suppressed during nicotine withdrawal

**Figure 5.**
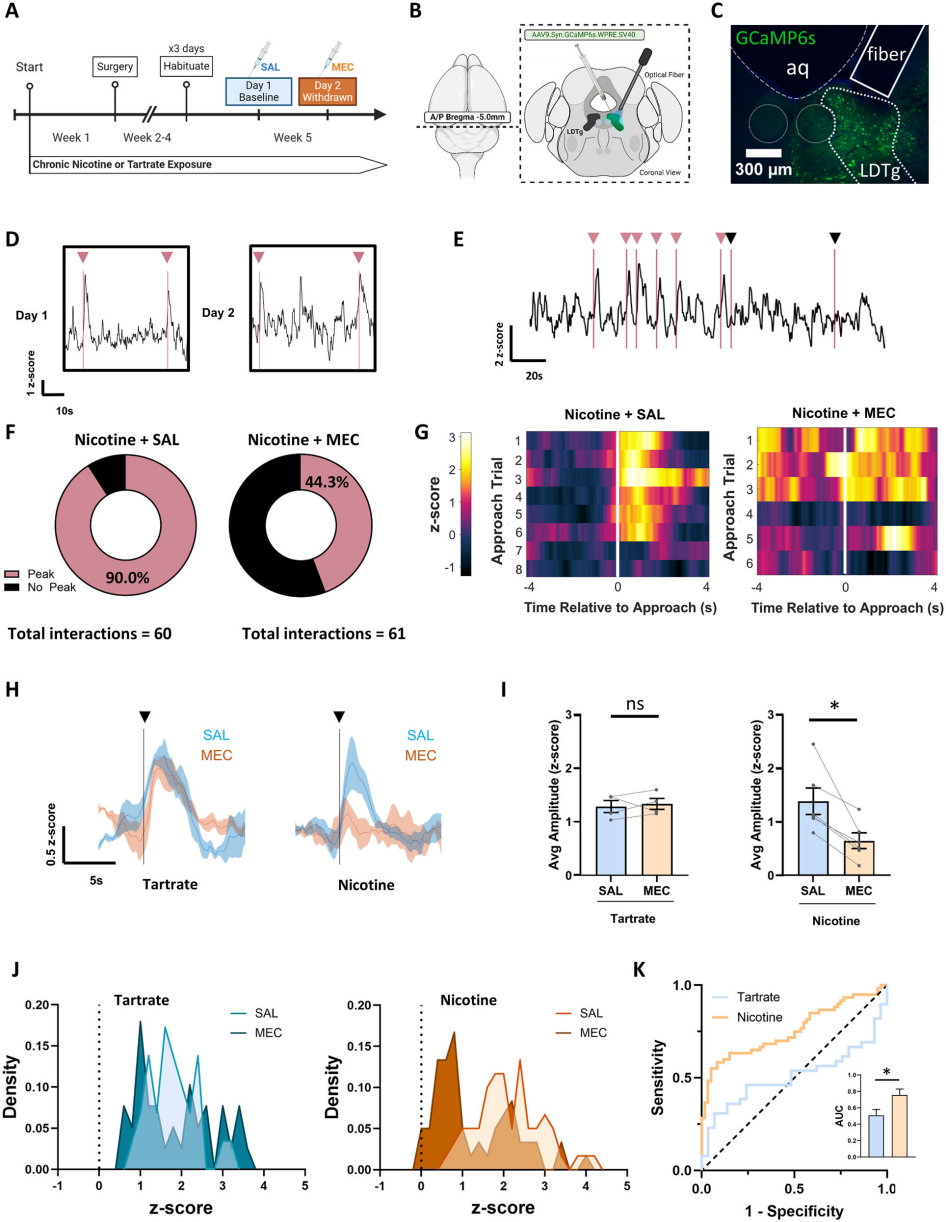
Nicotine withdrawal reduces LDTg neural activity during novel object interaction. ***A***, Timeline of nicotine drinking, surgery, and behavior. ***B***, Surgery schematic of pan-neuronal GCaMP6s expression in the LDTg and fiber-optic placement for cell body recording. ***C***, Histologic verification of viral expression and fiber location. ***D***, Sample trace of LDTg activity during novel object interactions (pink line with arrow; Day 1). Similar responses were recorded with a new novel object on Day 2. ***E***, Sample trace of LDTg activity with multiple novel object interactions, showing response variability. Most novel object interactions evoked increased activity (pink arrows), but some elicited no change (black arrows). ***F***, Proportion of novel object interaction corresponding to a peak in GCaMP6 signal (pink) compared with no detectable peak (black) in nicotine dependent (SAL) and nicotine withdrawn (MEC). ***G***, Representative heat map of GCaMP6s *z*-score responses to a series of novel object interactions (time 0) in a nicotine-dependent animal after injection of saline (SAL) or 2 mg/kg MEC. ***H***, Average traces with standard error aligned to novel object interaction (line with arrow) in nicotine-naive animals (tartrate, left, *N* = 4) after an injection of saline (SAL, blue) or 2 mg/kg MEC (orange). Recordings from nicotine-dependent animals (nicotine, right, *N* = 6) after an injection of saline (SAL, blue) or MEC (orange) precipitating a nicotine withdrawal state. ***I***, Average amplitude of peaks in GCaMP6 fluorescent signals immediately following all novel object interactions with each data point representing a single mouse before and after saline (SAL, blue) or 2 mg/kg MEC injection (MEC, orange). Within nicotine exposed animals (right, *N* = 6), the amplitude of peak following novel object interaction is decreased during MEC-precipitated nicotine withdrawal (two-way RM ANOVA, Holm–Sidak's multiple comparisons, SS = 0.7435, df = 1, *F* = 10.21, *p = 0.0030). There is no change in the average peak amplitude to novel object interaction in tartrate-exposed (nicotine naive, *N* = 4) mice, regardless of injection. ***J***, Probability density plot (binned by 0.2 increments) of average *z*-score 5 s post-novel object interaction for tartrate (blue, *N* = 4) and nicotine (orange, *N* = 6) exposed animals after an injection of saline (SAL) or MEC. ***K***, ROC curve analysis to assay discriminability of novel object evoked *z*-score GCaMP6s activity in the LDTg between groups (tartrate vs nicotine). ROC curves were calculated using Day 1 (SAL injection) and Day 2 (MEC injection), plotted against the line of equivalency (black dotted line). AUCROC analysis shows greater AUC in the nicotine (orange, *N* = 120) group than the tartrate (blue, *N* = 68) group (ROC area, *p < 0.0001, 95% confidence interval = 0.6699–0.8457).

Novel object interactions are salient stimuli that provide a time-locked method to probe associated neural activity in control mice and during nicotine withdrawal. To further investigate LDTg neural activity during novel experiences, we expressed the calcium sensor, GCaMP6s, in the nucleus under a pan-neuronal promotor with a fiber-optic cannula positioned above the nucleus (Fig. 5*A–C*). Mice were exposed to nicotine or tartrate control solution for 4 weeks, as described. We observed profound and highly reproducible increases in GCaMP6s signal in the LDTg in response to a novel object placed in a familiar environment, consistent with an increase in LDTg neural activity. In control mice, interactions with the novel object elicited a peak in GCaMP6s signal in ∼90% of the approaches to within 2 cm of the object (Fig. 5*E*,*F*). This effect was repeatable across multiple days, using different novel objects each day, allowing for within-animal comparison of novel object LDTg response after an injection of SAL (Day 1) or MEC (Day 2; Fig. 5*A*,*D*). During nicotine withdrawal, novel object interactions induced smaller peak amplitude responses in LDTg (Fig. 5*G–I*) and a decrease in response prevalence to ∼44% of interactions (Fig. 5*F*). To quantify this further, we calculated an individual average *z*-score for the 5 s post-novel object interaction in nicotine and tartrate groups (Fig. 5*J*). Probability density curves of *z*-score show more overlap of tartrate-exposed groups than between nicotine dependent (SAL) and nicotine withdrawal (MEC), as reflected by the ROC curves, showing the tartrate group is closer to the line of equivalency and the nicotine group is farther away (Fig. 5*K*). These data show that the nicotine withdrawal can be discriminated from control groups by the LDTg response to novel object.

### Novel object-induced changes in NAc DA levels exhibits variable dynamics in nicotine-naive, nicotine-dependent, and nicotine-withdrawn mice

**Figure 6.**
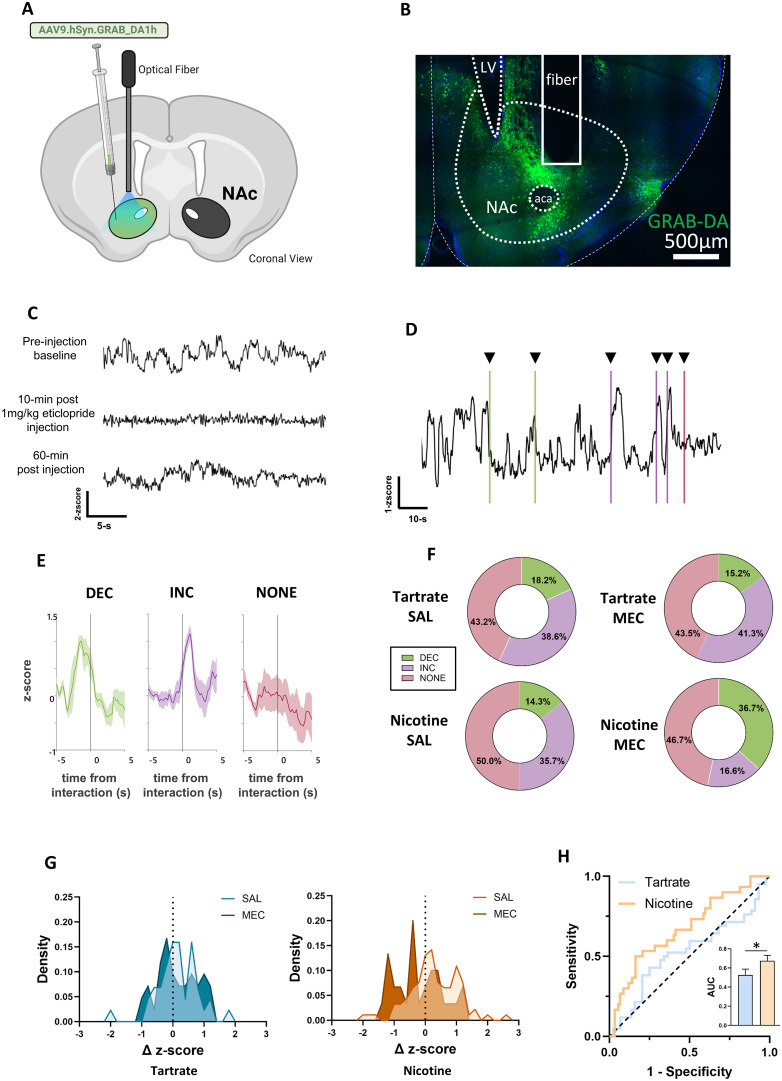
DA release to novel object interaction exhibits variable dynamics in nicotine-naive, nicotine-dependent, and nicotine-withdrawn mice. ***A***, Surgery schematic of GRAB-DA injection in the NAc and fiber placement to record changes in DA release. ***B***, Histologic verification of GRAB-DA expression in NAc and fiber placement above viral expression. ***C***, Sample traces of GRAB-DA signal before injection of eticlopride, 10 min post injection, and 60 min post injection. ***D***, Representative trace of GRAB-DA signal changes during several novel object interactions showing decreases in DA (green), increases in DA (purple), or no change in DA (pink). ***E***, Average GRAB-DA responses to novel object interaction with standard error grouped by DA response category. ***F***, Proportion of novel object response by group in tartrate (*N* = 5) and nicotine (*N* = 6) exposed animals after an injection of saline (SAL) or 2 mg/kg MEC. ***G***, Probability density plot (binned by 0.2 increments) of change (Δ) *z*-score (average *z*-score 5 s post-novel object interaction − average *z*-score 5 s pre-novel object interaction) for tartrate (blue, *N* = 5) and nicotine (orange, *N* = 6) exposed animals after an injection of saline (SAL) or MEC. ***H***, ROC curve analysis to assay discriminability of novel object evoked Δ *z*-score GRAB-DA signal between groups (tartrate vs nicotine). ROC curves were calculated using Day 1 (SAL injection) and Day 2 (MEC injection), plotted against the line of equivalency (black dotted line), with AUCROC analysis showing greater AUC in the nicotine (orange, *N* = 122) group than the tartrate (blue, *N* = 86) group (ROC area, *p = 0.0042, 95% confidence interval = 0.5619–0.7873).

Salience of novel object interactions is known to elicit elevated DA release in the NAc (Wingo et al., 2016). We observed that novel object interactions elicit robust increases in LDTg neural activity, and this likely contributes to enhanced DA release, as LDTg activation supports burst firing activity in DA neurons (Kohlmeier, 2013). As such, we hypothesized that reduction of LDTg activity during nicotine withdrawal could contribute to the associated hypodopaminergic conditions (Epping-Jordan et al., 1998; Stoker et al., 2008). To test this idea, we expressed a fluorescent DA sensor, GRAB-DA, in the NAc to monitor DA dynamics during novel object interactions (Fig. 6*A*,*B*). GRAB-DA is a modified DA D2 receptor, and we validated its DA sensitivity by monitoring the GRAB-DA photometry signal after an injection of 1 mg/kg eticlopride, a D2R antagonist. Transient increases in fluorescence intensity were inhibited 10 min after eticlopride injection (Fig. 6*C*). We also observed a partial recovery of GRAB-DA transients by 60 min postinjection (Fig. 6*C*). Somewhat unexpectedly, novel object interactions resulted in variable changes in NAc DA release. We observed three types of responses to novel object interaction (Fig. 6*D*,*E*). Similar to LDTg activity (Fig. 5*E–G*), we saw numerous examples of the increases in DA immediately following the novel object interaction (Fig. 6*D–F*). Additionally, we also observed interactions with no response along with cases where DA was elevated immediately before interaction, with the object interaction resulting in a rapid decrease (Fig. 6*D–F*). The proportion of novel object interactions that result in these three designations was relatively stable across control conditions, with slightly fewer increased DA responses during nicotine withdrawal (Fig. 6*F*). To quantify this, we calculated an individual change in *z*-score (Δ *z*-score = average *z*-score 5 s post-novel object interaction − average *z*-score 5 s pre-novel object interaction) for each novel object interaction in nicotine and tartrate groups. This metric yields large positive Δ *z*-scores for the increases in DA responses, large negative Δ *z*-scores for decreased DA responses, and minimal Δ *z*-scores for the interactions with no response (Fig. 6*E*,*G*). Probability density curves of Δ *z*-scores show more overlap of SAL and MEC tartrate-exposed groups than between nicotine control (SAL) and nicotine withdrawal (MEC), as reflected by the ROC curves with tartrate group is closer to the line of equivalency and the nicotine group is further away (Fig. 6*H*). Although the GRAB-DA responses vary, these data show that the nicotine withdrawal can be discriminated from control groups by the DA response to novel object.

### LDTg activity attenuates during nicotine withdrawal

**Figure 7.**
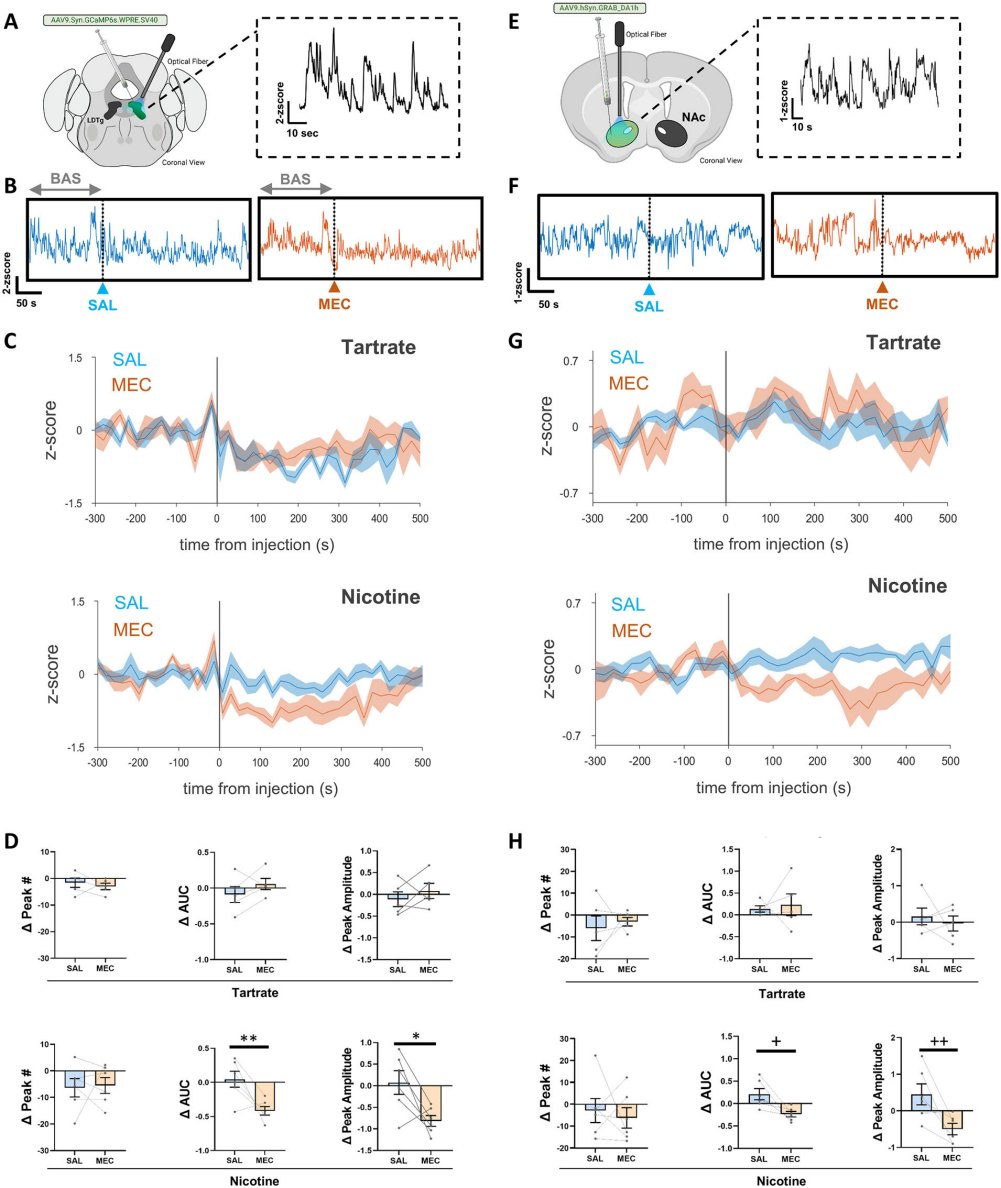
LDTg neural activity and DA release in the NAc are suppressed in nicotine withdrawal. ***A***, Surgery schematic of LDTg GCaMP6s injection and fiber implant with spontaneous activity (inset). ***B***, Representative LDTg GCaMP6s trace after 4 weeks of nicotine drinking before and after an injection of saline (SAL, blue) or 2 mg/kg MEC (MEC, orange). ***C***, Averaged LDTg GCaMP6 responses with standard error in tartrate drinking mice (top, *N* = 5) and nicotine drinking mice (bottom, *N* = 6) after an injection of saline (SAL, blue) or MEC (orange). ***D***, Spontaneous transient peaks in the GCaMP6 signal were detected during baseline and post injection of SAL or MEC in tartrate- or nicotine-pre-exposed mice. Change in the number of spontaneous transients (Δ Peak #) was relative to baseline was not different for nicotine-naive and nicotine withdrawal mice. The average area under the curve (AUC) for the baseline compared with postinjection (Δ AUC) did show a decrease of transient area in nicotine-withdrawn mice (two-way ANOVA, Sidak's multiple comparisons, SS = 0.5075, df = 1, *F* = 3.003, **p = 0.0088). Comparison of peak amplitudes revealed smaller events after MEC injection relative to saline and nicotine-naive mice (two-way ANOVA, Sidak's multiple comparisons, SS = 0.4234, df = 1, *F* = 7.284, *p = 0.0244). ***E***, Surgery schematic of NAc GRAB-DA injection and fiber implant with spontaneous activity (inset). ***F***, Representative NAc GRAB-DA trace after 4 weeks of nicotine drinking before and after an injection of SAL (blue) and an injection of 2 mg/kg MEC (orange). ***G***, Averaged NAc GRAB-DA data with standard error in tartrate drinking mice (top, *N* = 5) and nicotine drinking mice (bottom, *N* = 6) after an injection of SAL (blue) and MEC (orange). ***H***, Δ Peak #, Δ AUC, and Δ Peak amplitude parameters were calculated similar to the GCaMP6 signals (post injection − pre injection). There was no change in the number of DA peaks observed post injection for any of the treatment groups. AUC was decreased in nicotine drinking animals after an injection of MEC compared with SAL (two-way ANOVA, Sidak's multiple comparisons, SS = 0.8633, df = 1, *F* = 6.125, **p = 0.0304). Peak amplitude of DA transients was significantly decreased in nicotine drinking animals after MEC injection compared with SAL (two-way ANOVA, Sidak's multiple comparisons, SS = 1.774, df = 1, *F* = 5.873, **p = 0.0355) but not to tartrate controls.

IPN is activated during nicotine withdrawal (Zhao-Shea et al., 2013) and is known to send inhibitory projections to the LDTg (Wolfman et al., 2018). Therefore, we hypothesized that during nicotine withdrawal, LDTg activity would be suppressed. To test this, we exposed mice to nicotine or tartrate control solution for 4 weeks as described above. We expressed the intracellular calcium indicator, GCaMP6s, in the LDTg to record neuronal activity through a fiber-optic implanted directly above the nucleus (Fig. 7*A*). We monitored the activity of LDTg before and after an injection of SAL or MEC in nicotine-naive and nicotine-dependent mice (Fig. 7*B*,*C*). In nicotine-dependent mice, but not in tartrate controls, MEC injection decreased the amplitude and AUC of identified peaks without changing the peak frequency within the 5 min postinjection compared with the 5 min before injection (Fig. 7*D*). Average traces reveal an overall suppression of LDTg neural activity during MEC-induced nicotine withdrawal (Fig. 7*C*).

### Dopamine in the NAc is suppressed during nicotine withdrawal

Nicotine withdrawal leads to attenuated extracellular DA levels in the NAc as measured by microdialysis in rat and mouse (Hildebrand et al., 1998; Carboni et al., 2000; Rada et al., 2001; Zhang et al., 2012). Fluorescent DA sensors and fiber photometry provide a greater temporal resolution to monitor changes in subsecond time scales. In light of the suppression of LDTg activity that we observed during nicotine withdrawal, and the previous reports that LDTg activity is linked to increased burst firing in mesoaccumbens DA neurons, we expected that fiber photometry would show a suppression of DA release following nicotine withdrawal. Expressing the DA sensor, GRAB-DA, in the NAc (Fig. 7*E*) revealed a decrease in DA transmission after MEC-induced nicotine withdrawal (Fig. 7*F*,*G*). We observed decreased average peak amplitude and area under the curve (AUC) in the 5 min after MEC injection, compared with a 5 min baseline period before injection (Fig. 7*F–H*) similar to the responses observed in LDTg GCaMP recordings (Fig. 7*B*,*C*). MEC injection had no effect on peak amplitudes or AUC in tartrate control mice (Fig. 7*H*).

### IPN GABAergic neurons synapse differentially onto LDTg cell types

**Figure 8.**
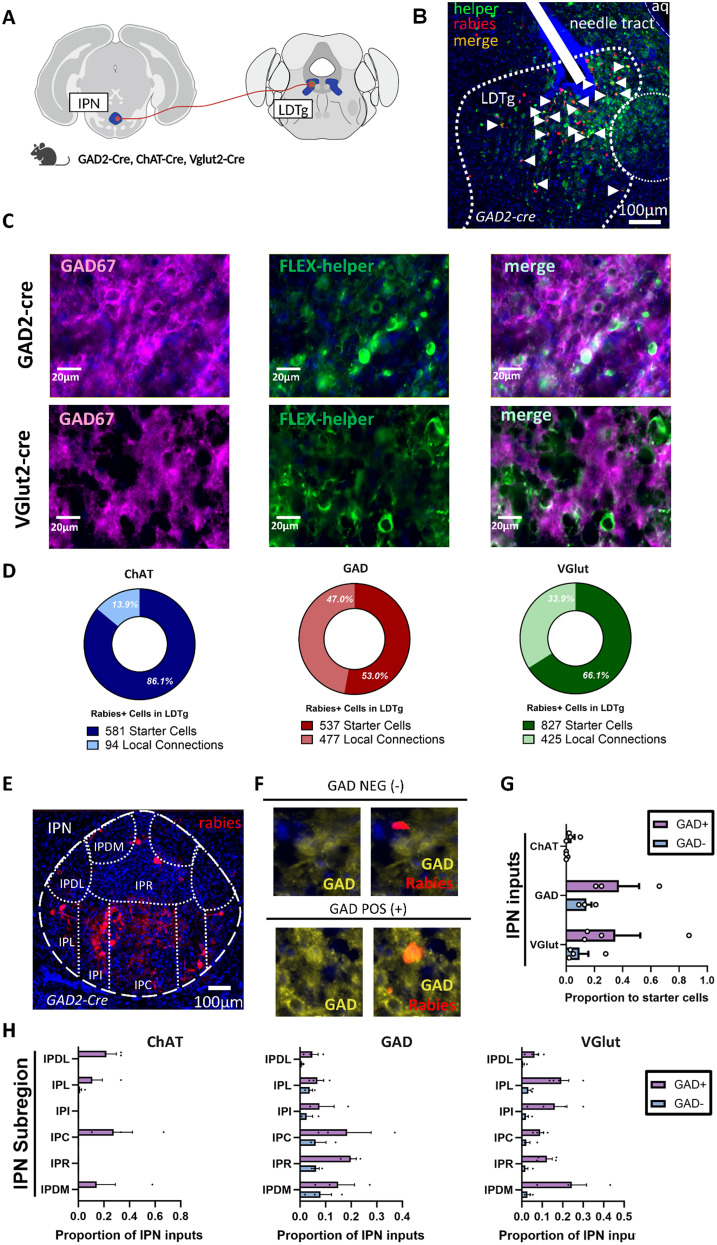
GABAergic IPN neurons synapse onto both GABAergic and glutamatergic LDTg neurons. ***A***, Surgery schematic of rabies backlabeling of IPN neurons synapsing to LDTg subtypes using GAD2-Cre (*N* = 3), ChAT-Cre (*N* = 3), and Vglut2-Cre (*N* = 4) mice. ***B***, GABAergic LDTg starter cells identified by overlap (orange) of helper virus (green) with rabies virus (red). White arrowhead points to representative starter cells identified within LDTg within a GAD2-cre mouse. Rabies only neurons (red alone) indicate local connections within LDTg to starter cells. ***C***, Cre-dependent helper virus expression is restricted by using cre mouse lines. Immunohistochemistry labeling GAD67 (purple) overlaps (light blue) with cre-dependent helper virus (green) in GAD2-cre mouse line (top panels). GAD67 (purple) does not overlap with helper virus (green) in Vglut2-cre mouse line. ***D***, Total rabies positive cells in LDTg divided into proportion of starter cells (rabies+, helper virus+) and local LDTg connections (rabies+, helper virus−). Rabies virus cells were identified in Chat-Cre, GAD2-cre, and Vglut2-cre mouse lines. ***E***, IPN rabies+ neurons are present in multiple IPN subregions. ***F***, IPN rabies+ neurons are identified as GAD+ or GAD− using GAD67 immunohistochemistry. ***G***, IPN inputs proportion to starter cells show IPN connections to all three LDTg subtypes (cholinergic, GABAergic, and glutamatergic) but shows more connections to GABAergic and glutamatergic LDTg neurons. ***H***, IPN subregions may show different patterns of distribution to LDTg subtypes.

The decrease in LDTg neural activity during nicotine withdrawal may be due to increased GABAergic drive to these neurons. GABAergic neurons in the IPN become active during nicotine withdrawal (Zhao-Shea et al., 2013) and send dense projections to the LDTg (Fig. 1*G*). Using ex vivo electrophysiology, our lab previously showed that the inputs from IPN to LDTg are GABAergic and that these connections are important in the aversive response to high acute doses of nicotine (Wolfman et al., 2018). However, it is currently unknown which cell types within the LDTg are inhibited by IPN inputs. To explore this question, we used rabies transsynaptic tracing to label monosynaptic functional GABAergic inputs from IPN to LDTg. A cre-dependent helper virus was expressed in the LDTg of either ChAT-cre, GAD-cre, or Vglut-cre mice to target cholinergic, GABAergic, or glutamatergic LDTg cell types, respectively (Fig. 8*A*). Immunohistochemistry (IHC) was used to verify that cre-driven virus expression is limited to the correct cell type (Fig. 8*B*,*C*). Two weeks after helper virus injection, we introduced a g-deleted rabies virus in the same location (Fig. 8*B*) to label pre- and postsynaptic partners. Starter cell populations were identified as the cells positive for both helper virus and rabies virus in the LDTg (Fig. 8*B*,*D*). Rabies positive cells in the LDTg that lacked staining for helper virus were indicative of local presynaptic inputs to the double-labeled neurons (Fig. 8*D*). A large proportion (∼53%) of LDTg rabies+ cells are local connections to GABAergic neurons within the LDTg (Fig. 8*D*). While we also observed local connections to cholinergic and glutamatergic LDTg cells, they were not as numerous as those that synapse onto GABAergic LDTg neurons, despite high numbers of starter cells (Fig. 8*D*). Monosynaptic inputs to LDTg from IPN were stained for GAD to identify subpopulation of GABAergic inputs (Fig. 8*E*,*F*). Unexpectedly, we found GABAergic IPN neurons project to both GABAergic and glutamatergic LDTg neurons, with a higher convergence index (number of input cells/starter cells) than cholinergic neurons (Fig. 8*G*). Most IPN projections to LDTg were GAD+ for all three LDTg starter cell populations (Fig. 8*G*). Examining the IPN subregions projecting to LDTg revealed distinct anatomical distributions of inputs to the three LDTg cell types (Fig. 8*E*,*H*). The distribution of IPN subregion inputs to LDTg reveal GABAergic IPN projections to the LDTg are synapsing largely on GABAergic and glutamatergic neurons, and these IPN neurons have cell bodies largely in more central IPN regions, such as IPC, IPR, and IPI. IPN GABAergic neurons only send sparse connections to the LDTg cholinergic population, with the projections coming from more lateral regions such as IPDL, IPL, and IPDM. While enhanced IPN activity during nicotine withdrawal leads to an overall suppression of LDTg activity, it will be interesting to explore the possible role of changes in LDTg GABA neural activity under these conditions.

## Discussion

These studies demonstrate a previously undefined functional role of LDTg in mediating nicotine withdrawal symptoms. Inhibiting IPN→LDTg GABAergic projections attenuates a range of nicotine withdrawal-associated behaviors, including place preference, somatic withdrawal signs, novel social odor responses, and novelty/open field exploration. Our data supports IPN as a central controller of these behaviors, which likely contributes to the observed mood disorders associated with nicotine withdrawal (Lee et al., 2019). How the IPN interfaces with limbic system circuitry to modulate these behaviors was largely unknown. Previously, our lab demonstrated that connections from the IPN to the LDTg are GABAergic and enhanced activity of these connections mediates aversion to high doses of nicotine (Wolfman et al., 2018). Extending these observations, we tested the consequences of enhanced withdrawal-associated IPN→LDTg activity on the activity of the target LDTg neurons, and ultimately the impact of these projections on NAc DA release. Our findings support the hypothesis that IPN activation during nicotine withdrawal suppresses DA signaling, contributing to withdrawal-associated behaviors. Lastly, we used rabies mono-transsynaptic tracing to analyze the GABAergic, cholinergic, and glutamatergic LDTg neuronal subtypes receiving IPN GABAergic inhibition and found the strongest innervation to LDTg GABAergic and glutamatergic cell types.

### IPN→LDTg GABAergic projections in nicotine withdrawal behavior

Nicotine relapse is higher than any other addictive drug, and environmental cues are important risk factors for relapse (Doherty et al., 1995; Stevenson et al., 2017). In our place preference testing, it is remarkable that optogenetic suppression of IPN→LDTg connections to relieve withdrawal symptoms did not lead to preference for that location unless the mice were undergoing nicotine withdrawal. These findings suggest that targeting IPN→LDTg GABAergic projections could reduce nicotine withdrawal and its effects on relapse with little to no abuse liability.

Somatic withdrawal signs assayed by different investigators vary widely, limiting the ability to compare results across studies. With the goal of increasing rigor, we combined the signs across multiple studies to avoid biasing results in favor of those signs yielding significant differences. We observed an increase in the duration of time spent performing somatic signs during a withdrawal state, but not the overall number. Our interpretation is that counting number of somatic signs skews results to signs with short durations, while underrepresenting behaviors that occur in long bouts. Interestingly, a recent study showed somatic signs decrease IPN activity, suggesting a role of these behaviors in silencing IPN activity (Klenowski et al., 2022).

Diminished hedonic behaviors, including decreased novelty seeking and social interactions, are important criteria for depression. To probe the affective state of the mice during nicotine withdrawal, we monitored preference to social odor, open field, and novel object exploration which measures depression-like behavior (Lehmann et al., 2013). Changes in hedonic state and response to novelty are common symptoms of nicotine withdrawal-induced depression and anxiety (Malkesman et al., 2010; Tadmor et al., 2017; Chellian et al., 2021). Our observations that suppressing IPN→LDTg activity reduced withdrawal-induced somatic and affective behavior are consistent with previous studies of IPN in nicotine withdrawal (Zhao-Shea et al., 2015). All together, our data suggest nicotine withdrawal-induced activation of the MHb→IPN pathway is relayed to the limbic system through the LDTg to dampen hedonia-related behaviors.

### Nicotine withdrawal alters normal LDTg response and DA release to novel object

We observed strong LDTg activation to novel object interaction using fiber photometry. Notably, we did not see this increase in abandoned approaches or in chamber wall interactions, even though locomotor behavior looks similar across these contexts. Interestingly, there were occasional novel object interactions without LDTg responses, and this circumstance increased in nicotine withdrawal. This is consistent with increased IPN GABAergic drive to the LDTg during nicotine withdrawal. Recently, the Tapper lab showed the IPN GABAergic neurons progressively increase activity with familiarity (Molas et al., 2017b), suggesting familiarity may recruit IPN GABAergic activation to suppress LDTg activity.

While the IPN may contribute to responses to novelty, previous research has also implicated the VTA, NAc, PFC, and hippocampus (Wingo et al., 2016). There is a strong connection between response to novelty and drug addiction that is likely mediated through the mesolimbic DA system (Wingo et al., 2016). Using fiber photometry to measure DA release, our results show that DA release in the NAc is highly variable to novel object interaction, with a shift toward decreased DA during nicotine withdrawal. While we cannot directly attribute the DA release to LDTg activity, it is possible that the reduced LDTg response to novel objects during nicotine withdrawal contributes to the decreased DA release. The observed complexity of NAc DA release is not unexpected. DA release is not fully understood, and evidence shows a range of DA responses in learning, reward, aversion, and motivation with variability across contexts (Berke, 2018; de Jong et al., 2019). Further investigations are needed to demonstrate LDTg necessity in DA response to novel objects and the contribution from other key modulators.

### Nicotine withdrawal alters LDTg activity and DA release

Recent studies have implicated the LDTg in the sensitizing effects and rewarding effects of addictive drugs (Forster et al., 2002; Nelson et al., 2007; Ishibashi et al., 2009). To date, the data implicating LDTg in addiction have relied on lesion-induced behavioral studies and electrophysiology following drug application (Kohlmeier, 2013). Here, we extended these studies using pan-neuronal GCaMP6s to monitor LDTg activity during nicotine withdrawal in vivo. Spontaneous activity changed in the size of peaks without a change in the number, which can indicate a decrease in the number of active neurons being recorded or a decrease in the coordinated activity of the same population of neurons.

The LDTg provides strong input to midbrain DA neurons that project to the NAc (Oakman et al., 1995; Holmstrand and Sesack, 2011; Kohlmeier, 2013). LDTg activation increases VTA DA cell burst firing, decreases VTA GABAergic firing, and produces place preference (Forster and Blaha, 2000; Dautan et al., 2016; Steidl et al., 2017; Coimbra et al., 2021). Monitoring DA release in NAc during nicotine withdrawal, we observed a decrease in peak amplitude and peak AUC without a change in peak number. Fiber photometry provides greater temporal specificity than previous microdialysis studies of DA release (Hildebrand et al., 1998; Zhang et al., 2012). Decreased DA during drug withdrawal is hypothesized to arise from increased inhibition of VTA DA neurons, but our data suggest a potentially important contribution of reduced LDTg activity due to IPN activation.

### The IPN sends GABAergic synaptic connections differentially to cell types in LDTg

The LDTg GABAergic, glutamatergic, and cholinergic neurons are largely distinct cell types with limited neurotransmitter coexpression (Wang and Morales, 2009). Here, we observed the IPN subregion-specific connections to the LDTg through rabies-mediated monosynaptic tracing and identified the GABAergic nature of these inputs using IHC staining. Within the LDTg, we see a greater number of local synaptic connections to GABAergic and glutamatergic LDTg neurons than to cholinergic neurons. One interpretation of this result is that GABAergic and glutamatergic cells receive more synaptic connections from local interneurons. Another interpretation is that cholinergic local connections are coming from cholinergic neurons in other nuclei, while GABAergic and glutamatergic cells receive local synaptic connections from cell types different than the starter cell population, and this effect was masked by the helper virus expression.

There is strong topological organization of MHb to IPN connections. Ventral MHb (vMHb) innervates the majority of IPN with excitatory connections to the most central and dorsal regions, while the dorsal MHb (dMHb) innervates the most lateral regions with glutamatergic, substance P, and Neurokinin B signaling (Ables et al., 2023). This topography has important functional implication with vMHB more specifically regulating drug addiction, withdrawal, anxiety, and depression (Quina et al., 2017). IPN functions also vary by subregion with the greatest c-fos activation during nicotine withdrawal within the more central regions (Zhao-Shea et al., 2013, 2015; Matos-Ocasio et al., 2021). Our results suggest that GABAergic neurons from IPN to LDTg are more likely involved in nicotine withdrawal. Interestingly, there is evidence that LDTg sends reciprocal GABAergic connections to the IPN, which have been proposed to have an overall disinhibition and temporally coordinated oscillatory activity (Caputi et al., 2013; Bueno et al., 2019). On the other hand, cholinergic LDTg neurons receive connections from the lateral IPN, which are less implicated in nicotine withdrawal.

In summary, our findings highlight a previously undefined role of IPN projections to the LDTg in nicotine withdrawal and a potential target for treating nicotine addiction. We found that IPN inputs to LDTg contribute to MEC-precipitated withdrawal, a robust and reproducible method with behavioral efficacy in both male and female mice. We revealed a newly defined role of the LDTg in novelty exploration. Exploring this further will help resolve the neural basis of novel versus familiar to distinguish stress from neophobia. Additionally, while we observed withdrawal-induced changes in NAc DA release, we know that DA can contribute to a range of distinct behaviors, depending on the NAc location and postsynaptic targets. Lastly, our study extensively highlights specific connections from IPN→LDTg, and much more investigation is needed to appreciate both pre- and postsynaptic modifiers of this circuitry.

## Data Availability

Primary data and modified versions of our data analysis software are available upon request.
